# A rare presentation of myxofibrosarcoma as a Pancoast tumor: a case report

**DOI:** 10.1186/s13256-017-1223-5

**Published:** 2017-03-07

**Authors:** Vasa Jevremovic, Adnan Yousuf, Zulfiqar Hussain, Amer Abboud, Edgar G. Chedrawy

**Affiliations:** 1 Department of Surgery, Weiss Memorial Hospital, Chicago, IL USA; 2 Department of Oncology, Meadville Medical Center, Meadville, PA USA; 3 Department of Pathology, Weiss Memorial Hospital, Chicago, IL USA; 4 Department of Surgery, University of Illinois at Chicago, Chicago, IL USA

**Keywords:** Case report, Myxofibrosarcoma, Pancoast syndrome, Horner’s syndrome, Malignant fibrous histiocytoma

## Abstract

**Background:**

Myxofibrosarcoma is an aggressive soft tissue neoplasm, classified as a variant of malignant fibrous histiocytoma. Most often, it occurs in middle to late adult life peaking in the seventh decade and involving the lower extremities (77%), trunk (12%), and retroperitoneum or mediastinum (8%). We report the first case of thoracic myxofibrosarcoma presenting as a Pancoast tumor.

**Case presentation:**

A 48-year-old non-tobacco smoking African-American man presented with a slow-growing mass in his neck along with 11 kg weight loss over 9 months. A review of his systems was positive for hoarseness and lowgrade intermittent fever without any shortness of breath or cough. A physical examination revealed a mass on the left side of his neck superior to his sternoclavicular joint measuring 3 × 3 × 1 cm. He had ptosis and miosis of his left eye. His breath sounds were decreased and coarse at the left apex. A neurological examination revealed 3/5 strength in his left upper arm. The remainder of the physical examination was unremarkable.

Ultrasound of his neck showed an ill-defined heterogeneous mass lateral to his left thyroid lobe. A computed tomography scan of his chest showed a large multiloculated pleural-based mass in his left lung surrounding the adjacent neurovascular structures. A percutaneous biopsy was non-diagnostic. Subsequently, he underwent a left thoracotomy with biopsy. The mass extended from his anterior mediastinum medially at the level of the pulmonary trunk, superiorly into the superior sulcus and posteriorly into his chest wall. Surgical pathology confirmed the diagnosis of myxofibrosarcoma.

**Conclusions:**

Here we present a case of Pancoast tumor with myxofibrosarcoma as the underlying etiology. Pancoast syndrome generally entails an infiltrating lesion in the superior sulcus presenting with upper extremity pain, atrophy of the hand muscles, and Horner’s syndrome. The differential diagnosis of Pancoast syndrome includes inflammatory and infectious etiologies, as well as neoplasms of benign and malignant nature. Of the neoplasms implicated, the most common are non-small cell lung carcinomas; myxofibrosarcoma presenting as a Pancoast tumor has not been reported in the literature.

## Background

Myxofibrosarcoma (MFS) is an aggressive soft tissue neoplasm classified as a variant of malignant fibrous histiocytoma (MFH). On histologic examination it is composed of both fibroblastic and histiocytic elements. Most cases occur in middle-to-late adult life with a peak incidence in the seventh decade [1, 2]. They are predominantly encountered in the lower extremities (77%), trunk (12%), and retroperitoneum or mediastinum (8%) [1–3]. To the best of our knowledge only 19 cases of head and neck MFSs have been described in the literature, making it exceedingly rare [1]. For these sarcomas, the best approach to treatment is complete surgical resection [1]. Of all case presentations, none have had Pancoast syndrome as the initial manifestation. We are reporting the first case of MFS presenting as a Pancoast tumor.

Pancoast syndrome involves an infiltrating mass at the apex of the lung [4, 5]. Clinical manifestation is related to lesion location in the superior sulcus, with ipsilateral shoulder and arm pain, and weakness and atrophy of the ipsilateral hand muscles all due to brachial plexus compression or invasion. Commonly, Pancoast tumors also exhibit concurrent Horner’s syndrome along with other signs and symptoms of malignancy.

The majority of superior sulcus tumors are non-small cell lung carcinomas (NSCLCs) [5, 6]. Other etiologies less common for Pancoast syndrome include inflammatory, infectious, and neoplastic entities [4–6]. The treatment modalities for Pancoast tumor are determined by the specific nature of the neoplastic process in question. Surgical intervention is indicated in the majority of these instances. The presence of Horner’s syndrome and advanced disease are associated with a dire prognosis [4, 6].

## Case presentation

**Fig. 1:**
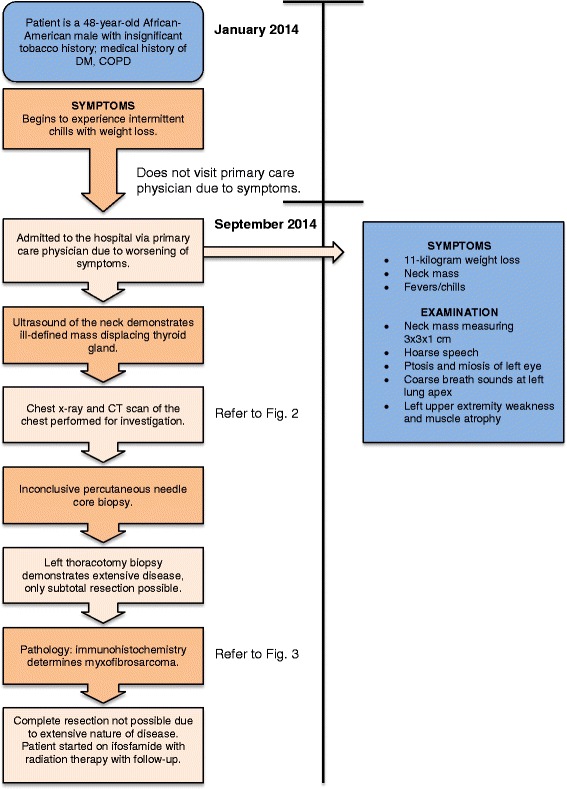
Timeline of case presentation. *COPD* chronic obstructive pulmonary disease, *CT* computed tomography, *DM* diabetes mellitus

A 48-year-old African-American man with no significant tobacco history presented with a slow-growing mass in his neck and 11 kg weight loss over a 9-month period (Fig. 1). On further history, he reported left arm pain and weakness, intermittent fever with chills, and had denied any dyspnea, shortness of breath, or cough. His past medical history was significant for diabetes mellitus, chronic obstructive pulmonary disease, and nerve impingement of his left shoulder. On physical examination, a mass was noted on the left side of his neck just superior to the sternoclavicular joint measuring 3 × 3 × 1 cm. He had ptosis and miosis of his left eye. His speech was hoarse, indicative of recurrent laryngeal nerve involvement. His breath sounds were decreased and coarse at the left apex. The strength of his extremities exhibited 3/5 weakness in his left arm, with the remainder of his limbs normal. There was positive muscle atrophy of his left upper extremity. Otherwise, the remainder of the physical examination was benign in nature.

**Fig. 2:**
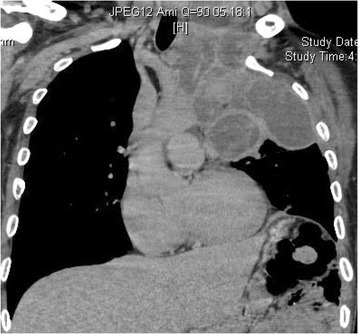
Coronal contrast-enhanced computed tomography of the neck and chest demonstrating a large multiloculated mass

An ultrasound performed on the neck mass showed an ill-defined heterogeneous mass lateral to his left thyroid lobe, resulting in mild mass effect. Further investigation was warranted with radiological studies including chest X-rays and a computed tomography (CT) scan of his chest. A chest X-ray exhibited a mass extending from the hilum of his left lung with gross deviation of his trachea and elevation of his hemidiaphragm. A CT scan of his chest showed a large multiloculated pleural-based mass in his lungs surrounding his left common carotid artery, left subclavian artery, which interfaced broadly with the aortic arch and extended into the left neuroforamina of C4/C5 through T1/T2 (Fig. 2).

**Fig. 3:**
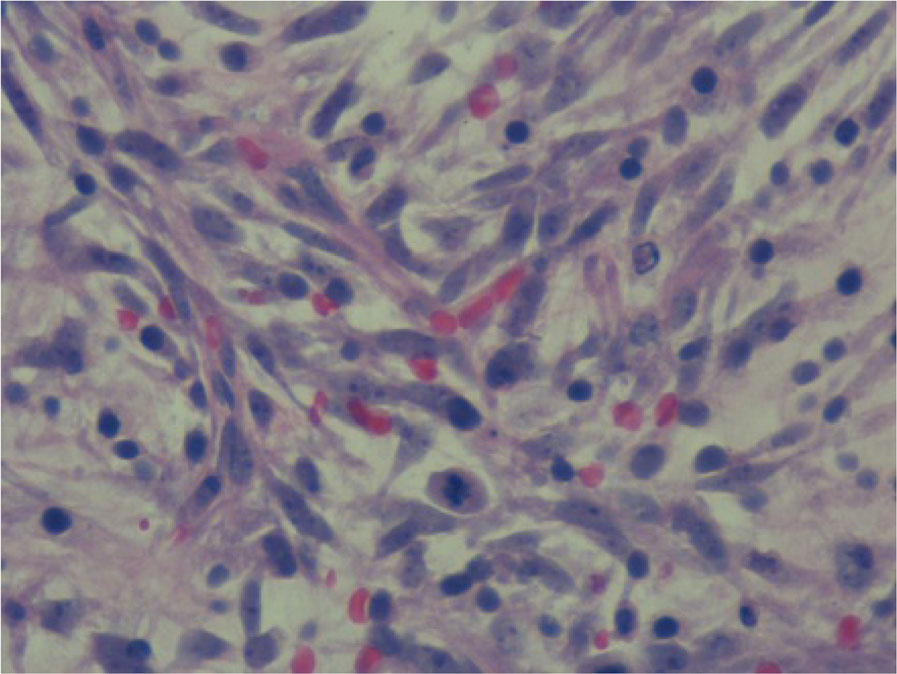
Hematoxylin and eosin stain: predominance of spindle-shaped cells and extravasation of erythrocytes characteristic of myxofibrosarcoma of high grade

Percutaneous needle core biopsy was performed on his neck and showed fibrohistiocytic tissue with mild chronic inflammation but was negative for malignancy. A left thoracotomy with biopsy was performed of the pleura and of the upper and middle lung lobes of his left lung evidencing a mass extending from the anterior mediastinum medially at the level of his pulmonary artery trunk superiorly into the superior sulcus and posteriorly into his chest wall. Tissue samples were obtained, all demonstrating a gross appearance of a pink to yellow gelatinous tissue for which frozen sectioning revealed soft tissue malignancy. Hematoxylin and eosin staining demonstrated a predominance of hyperchromatic spindle-shaped cells with acidophil cytoplasm (Fig. 3). This is consistent with high-grade sarcomas [7] typically with disorganized pleomorphic cells that can also include histiocytes and giant multinucleated cells [8]. A panel of immunohistochemical stains was performed of which only Wilms tumor protein (WT1), vimentin, and Ki67 were positive. This finding ruled out carcinoma, mesothelioma, melanoma, vascular tumor, synovial sarcoma, and Kaposi’s sarcoma as possible etiologies. Overall, immunohistochemistry revealed a high-grade undifferentiated myxoid sarcoma confirmed by the Mayo Clinic as fibromyxosarcoma versus MFS.

## Discussion

Head and neck presentation of MFS is extremely rare. To the best of our knowledge, of the 19 cases of head and neck MFS reported in the literature, our case is the first presenting as a Pancoast tumor. Pancoast syndrome entails superior sulcus infiltration presenting with upper extremity pain and atrophy of the hand muscles, commonly with concurrent Horner’s syndrome [5, 6]. The differential diagnosis of a Pancoast syndrome includes inflammatory etiologies such as sarcoidosis and granulomatosis with polyangiitis, infectious etiologies such as tuberculosis and aspergillosis, and neoplasms of benign and malignant nature [5, 6]. Of the neoplasms implicated, the most common are NSCLCs; MFS presenting as a Pancoast tumor has not been reported in the literature [5].

MFS is characterized by nodular growth of spindle-shaped or stellate-shaped fibroblastic cells in a myxoid matrix [2, 9]. This entity has been divided into four grades pertaining to cellularity, atypia, and extent of myxoid component [1–3, 9]. High-grade MFS (HGMFS) demonstrates a dominance of patternless histiocyte-like cells with pleomorphism in a minimal myxoid matrix [9]. Characteristic features differentiating this grade of lesion include areas of necrosis with lymphocytic infiltration, numerous and atypical mitotic figures, multinucleated giant cells, and extravasation of erythrocytes [2, 3, 9]. HGMFS lesions are frequently encountered in intramuscular and subfascial anatomy as opposed to the superficial (subcutaneous or dermal) manifestations of lower-grade lesions [2, 3, 9]. The differential diagnosis for HGMFS includes lesions of lower grade, nodular fasciitis, myxoma, extraskeletal myxoid chondrosarcoma, and pulmonary artery sarcomas [2, 3, 7, 10]. The characteristic features of our case presentation favored HGFMS as a diagnosis over other etiologies and lesions of lower grade.

Mentzel, Angervall, Merck, and their colleagues have previously described the relation between histologic grade of MFS and rate of local recurrence [2, 9]. The local recurrence of HGMFS had been documented as 55%, 64%, and 61% by Mentzel, Angervall, and Merck, respectively [2, 9]. The patient in our case report did not demonstrate any metastatic lesions. Mentzel, Angervall, and Merck had similarly described metastatic rates as 29%, 27%, and 38%, respectively [2, 9]. HGMFS has a less favorable outcome when compared to lower grades, with a mean survival of 2 months without intervention and extension to 10 months with tumor resection [11, 12]. As such, complete surgical resection is the mainstay treatment for MFS. Unfortunately, there is little literature regarding combination radiochemotherapy for cases of MFS that have suboptimal or no surgical excision [1, 13]. Although the tumor was evidently a suboptimal candidate for complete surgical resection, less invasive modalities of histological diagnosis were not employable. Thus, a left thoracotomy was opted for with the goal of definitive tissue diagnosis and resection of safely removable tumor mass. Unfortunately, an insufficient margin of resection was the result due to the extensive involvement of critical structures. Mediastinal excision of the mass measuring 6 × 6 cm was performed with partial decortication. More studies are necessary to investigate the efficacy of adjuvant radiochemotherapy for head and neck MFS, with a focus on cases where surgical management is difficult to attain. Although this entity is difficult to treat, follow-up entails assessing symptom progression and serial imaging [1, 9, 13].

Our patient had requested transfer of care to an oncology center with more proximity to his home address. They had begun treatment with high-dose ifosfamide. The first cycle of treatment was well tolerated, resulting in an apparent decrease in tumor size. He had returned for a second cycle of ifosfamide with an initial dose of high-intensity radiation with the high-dose spatially-fractionated radiation (GRID) approach followed by additional doses with conventional radiation.

## Conclusions

In conclusion, MFS is a relatively uncommon malignancy and has not been described as a Pancoast tumor in the medical literature. Surgical resection may result in insufficient margins when extensive invasion within the superior sulcus and critical structures of the thorax is involved. Given that this management is the mainstay for MFS, more research is necessary with regards to adjuvant therapies that can improve prognosis.

## Abbreviations

CT: Computed tomography
HGMFS: High-grade myxofibrosarcoma
MFH: Malignant fibrous histiocytoma
MFS: Myxofibrosarcoma
NSCLC: Non-small cell lung carcinoma
